# Standardization of organoid culture in cancer research

**DOI:** 10.1002/cam4.5943

**Published:** 2023-04-20

**Authors:** Changchun Zhou, Yuanbo Wu, Zeyu Wang, Yanli Liu, Jiaqi Yu, Weiping Wang, Sunrui Chen, Weihua Wu, Jidong Wang, Guowei Qian, Aina He

**Affiliations:** ^1^ Biobank, Cancer Research Center, Shandong Cancer Hospital Shandong First Medical University, Shandong Academy of Medical Sciences Jinan Shandong China; ^2^ Department of Ultrasound Yangxin County People's Hospital Huangshi Hubei China; ^3^ Department of Gastrointestinal Surgery Renji Hospital Affiliated to Shanghai Jiaotong University School of Medicine Shanghai China; ^4^ Department of Pharmacology and Pharmacy, Dr. Li Dak‐Sum Research Centre The University of Hong Kong Hong Kong China; ^5^ Shanghai OneTar Biomedicine Shanghai China; ^6^ Department of Oncology Shanghai Sixth People's Hospital Affiliated to Shanghai Jiaotong University Shanghai China

**Keywords:** extracellular matrix, organoid culture system, standardization, tumor microenvironment, tumor organoids

## Abstract

Establishing a valid in vitro model to represent tumor heterogeneity and biology is critical but challenging. Tumor organoids are self‐assembled three‐dimensional cell clusters which are of great significance for recapitulating the histopathological, genetic, and phenotypic characteristics of primary tissues. The organoid has emerged as an attractive in vitro platform for tumor biology research and high‐throughput drug screening in cancer medicine. Organoids offer unique advantages over cell lines and patient‐derived xenograft models, but there are no standardized methods to guide the culture of organoids, leading to confusion in organoid studies that may affect accurate judgments of tumor biology. This review summarizes the shortcomings of current organoid culture methods, presents the latest research findings on organoid standardization, and proposes an outlook for organoid modeling.

## 1 INTRODUCTION

Tumor heterogeneity is an obvious obstacle to effective treatment of cancer, it exhibits distinct phenotypes and evolves during cancer development and progression.^1^, ^2^ Successful implementation of precision medicine depends on whether we systematically define tumor heterogeneity and simulate it in vitro. Tumor heterogeneity is currently divided into inter‐ and intra‐tumor heterogeneity. Inter‐tumor heterogeneity includes spatiotemporal and extracellular diversity from different patients with alterations caused by different etiological and environmental factors, while intra‐tumor heterogeneity describes the variation of different tumor, stromal, and immune cell populations within the same tumor specimen.^3^, ^4^ Dissecting heterogeneity between or within tumors will facilitate tumor research, which requires standardization and reproducibility of primary tumor cultures.^5^, ^6^

Organoid culture has become a representative platform for both basic and translational cancer research.^7^ Tumor organoids are of great value in recapitulating histopathological, genetic, and phenotypic characteristics of patient‐derived tumor tissue.^8^ This technique has constructed several cancer organoid biobanks, including pancreas, prostate, ovary, bladder, liver, breast, lung, esophagus, stomach, endometrium, kidney, and brain cancers.^9^, ^10^, ^11^, ^12^, ^13^, ^14^, ^15^ Organoids more accurately reflect the characteristics of the original tumor than traditional cell lines, and they can mimic tumor microenvironment (TME)‐cell interactions by co‐culture with non‐tumor cells.^10^, ^16^ Tumor organoids have been used in precision medicine as part of a high‐throughput screening platform by testing their sensitivity to anticancer drugs.^17^, ^18^, ^19^, ^20^

Organoids have great potential as a tumor model, but the criteria for organoid culture and the definition of successful culture are not yet clear, which leads to technical variations in bench work and affects the judgment of biological heterogeneity in tumors.^11^, ^21^ These variations may include inconsistent tissue dissociation, unclear medium formula, and different matrix used for the organoid system.^22^, ^23^ Tumor organoids are mainly generated from epithelial tumors, few studies pay attention to non‐epithelial tumors (e.g., glioblastoma [GBM] and chordoma).^24^, ^25^ Current tumor organoids typically include only tumor cells and lack TME to support long‐term cell culture.^22^ Due to the lack of a suitable platform to mimic cell–cell interactions, the mechanism of the extracellular matrix (ECM) in driving organoid phenotype and drug sensitivity is largely unexplained.^26^, ^27^

In this review, we discuss the limitations of current organoid culture system, highlight the recent innovative progress in culture standardization, and propose a development direction for organoid modeling.

## 2 LIMITATIONS OF CURRENT ORGANOID CULTURE SYSTEM

**FIGURE 1:**
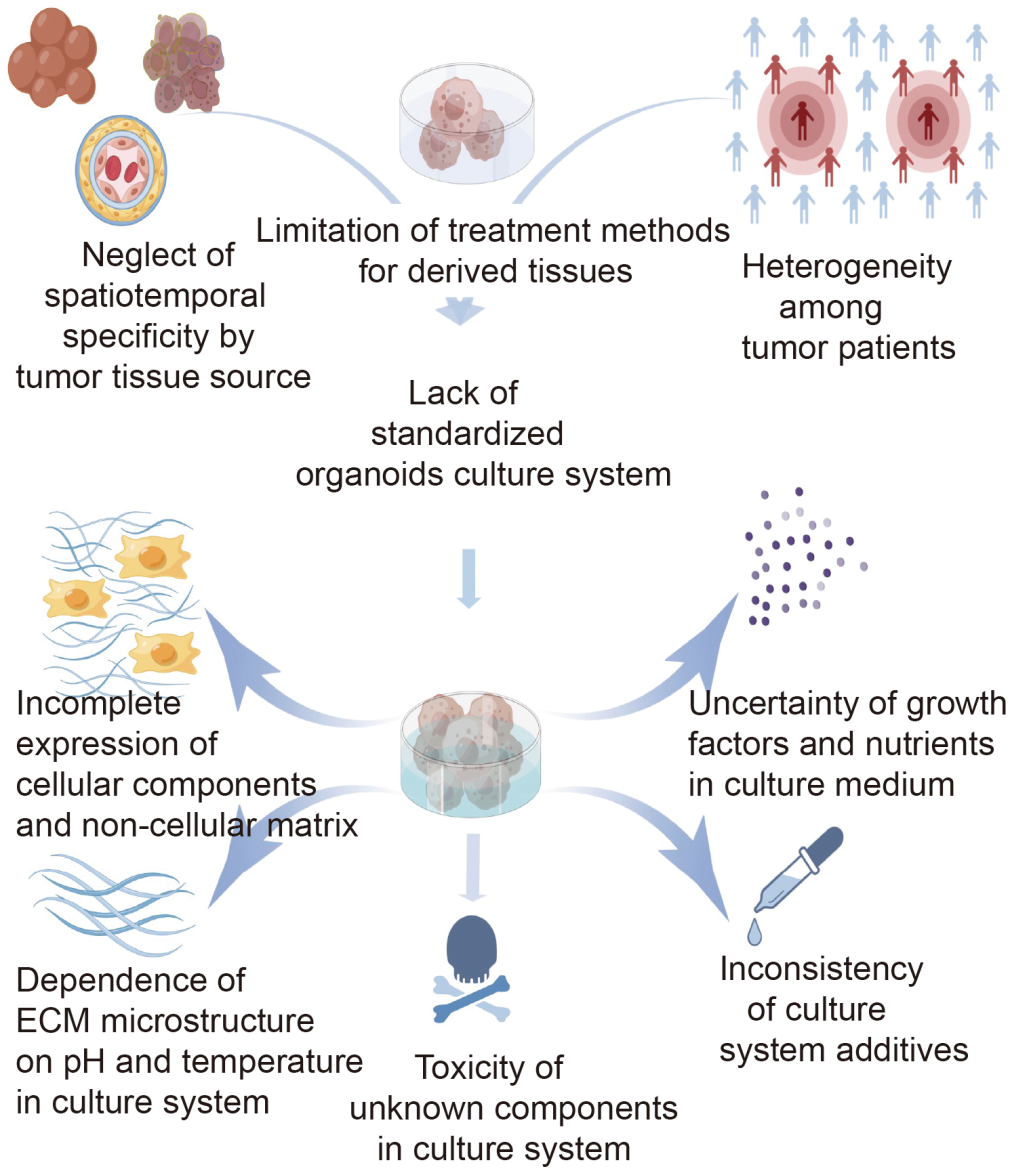
Lack of standardization of current organoid culture system. Inter‐ and intra‐tumor heterogeneity make organoid culture more complex. The existing organoid culture system is more artificial, cellular components, growth factors, and nutrients within the culture medium are not fully interpreted. The figure is drawn by Figdraw (www.figdraw.com).

The protocols of organoid construction vary widely. Although each culture method claims to be appropriate for the research of tumor biology and therapeutic efficacy, the current culture techniques have not been unified, which limits their preclinical application. Here, we describe the shortcomings and pitfalls brought by technical bias to organoid culture (Figure 1).

### 2.1 Limitations of tumor tissue sampling

In order to capture tumor heterogeneity using organoids, the first critical step is to obtain original samples that reflect the temporal and spatial diversity of tumors. Sources of tissues usually include solid or liquid biopsy, surgical resection, and rapid autopsy.^17^, ^18^, ^28^, ^29^ At present, organoid cultures are mainly derived from a single‐point biopsy or surgical resection, circulating tumor cells or effusive cells of primary tumors and metastasis. Each of them is incompetent to reflect tumor heterogeneity.^30^, ^31^ Assuming that the collected tumor sources are not representative of the entire tumor, the constructed organoids cannot fully reflect drug response, which may lead to failures in drug screening and biomarker discovery.

Rapid growth of tissue‐derived healthy cells may contaminate tumor organoids, limiting their construction and application in tumor biology.^10^, ^18^, ^29^, ^30^ A study examining the purity of lung adenocarcinoma organoids in 59 patients showed that 58% of the organoids overgrew due to contamination of healthy airway cells.^32^ There are many restrictions on the acquisition and collection of primary tissues, which are beyond the control of the laboratory. Treatment of tumor patients prior to tissue collection may produce different organoid phenotypes and drug responses in vitro.

### 2.2 Limitations of primary tissues processing methods

Primary tissues are dissociated into individual cells by enzymatic or mechanical methods, and complete dissociation is conducive to organoid culture. Enzymatic hydrolysis may lead to off‐target cleavage effects, and enzymes are tissue‐specific.^33^ Enzymatic dissociation of tissues produces cell populations with different sizes, ranging from a single cell to a cell cluster with a diameter of about 100 μm. Complete tissue dissociation often disrupts cell–cell interactions and expands the negative selection of non‐tumor cells.^33^ Another method of tissue processing is to chop up tumor tissues directly and perform three‐dimensional (3D) culture on intact millimeter‐scale tumor fragments. Compared with enzymatic dissociation, this approach preserves the native structure of tissue and TME cellular components, which modulate organoid formation and phenotype. Mechanical cutting produces non‐reproducible tissue fragments and brings non‐uniform environment of encapsulated cells, including uneven oxygen concentrations and nutrient gradients in larger fragments. Blunt tissue cutting can also damage tumor samples, further reducing the number of living cells needed for successful organoid culture.^34^, ^35^

### 2.3 Limitations of organoid culture environment

#### 2.3.1 Limitations of organoid culture medium

Since most organoid models are composed of pure tumor cell populations, it is necessary to understand the stem cell niche and specific factors that allow stem cells to self‐renew and proliferate. The medium should be supplemented with growth factors required for tumor cell growth (usually secreted by TME cells in vivo).

Some purified recombinant protein factors, due to poor solubility and insufficient long‐term storage stability, reduce their activity, limiting accurate modeling of specific tumor niches. Extensive use of conditioned medium derived from mammalian cells (e.g., containing Wnt‐3a, Noggin or R‐spondin) reduces the cost of generating organoids and improves their culture.^36^ However, conditioned medium suffers from batch‐to‐batch variability in their composition and contains unknown extra factors that may have unclear impact on organoid phenotype, which may affect the reproducibility of experiment. The inclusion of animal‐derived serums in the medium may also lead to non‐standardization of organoid modeling. Although fetal bovine serum has been widely used, the exact composition of serum remains unclear and may lead to inconsistent organoid growth.^37^ Animal‐derived serums are heterogeneous, it is of concern that fetal bovine serums have significant and unknown effects on organoid culture and phenotype.^38^ Seino et al. found that conditioned medium containing fetal bovine serum failed to produce healthy pancreatic organoids over a long period of time.^10^

#### 2.3.2 Limitations of organoid ECM

ECM is an organized network containing a variety of proteins and polysaccharides with certain structure and biochemical functions. Compared with highly regulated ECM around healthy tissues, structure, and function of ECM in tumors are transformed and disordered.^39^, ^40^ Comprehensive understanding of tumor–ECM interaction requires robust and suitable models to control stromal regulation. At present, there is no comprehensive study using tumor organoids to mimic intra‐ or inter‐tumor ECM heterogeneity, and only a few organoid models have explored the role of ECM in tumor pathogenesis and anti‐tumor response after treatment. Most in vitro organoids rely on animal‐derived scaffolds with unclear composition and poor tunability, which makes it difficult to standardize culture operation and limits the understanding of organoid–ECM interactions.^41^

Engelbreth‐Holm‐Swarm (EHS) matrix has been a common substrate for organoid culture.^42^ After extraction from mouse tumors, some ECM proteins, mainly laminin (~60%) and collagen IV (~30%), were retained in the recombinant EHS matrix, which provided structural and biological support for primary cell culture.^43^ EHS matrix is widely used in organoid research because it provides abundant tumor ECM components, growth factors, and cytokines.^44^ Generation of EHS matrix is simple, involving the steps of laminin self‐assembly and endogenous nestin‐1 mediated cross‐linking of laminin with collagen. EHS matrix derives from animals, varies widely from batch to batch, and contains heterogeneous impurities that are poorly understood. Matrigel contains more than 14,000 peptides and 2000 proteins, many of which may alter the phenotype of tumor cells.^44^ The biochemical and physiological characteristics of EHS matrix are not well understood, which makes it impossible to effectively present the ECM characteristics of primary tumors. Higher matrix stiffness is required for healthy tissue culture (e.g., human mammary tissue ~400 kPa vs. invasive breast cancer tissue ~5 kPa).^45^ The viscosity of the EHS matrix limits its use in large‐scale pharmaceutical applications because of the difficulty of handling liquid substrates. These restrictions have hindered the study of mechanism discovery, and huge costs have hindered high‐throughput drug screening. Even the shortcomings of EHS in material composition, repeatability and automation have been alleviated, there are more animals needed to expand the scale of EHS to drug screening, which is ethically problematic.

Another common matrix used for organoid culture is collagen matrix. Extreme desmoplastic response in solid tumors is usually related to the deposition and remodeling activity of collagen (usually I–IV).^46^ Type I collagen matrix has emerged in organoid culture as a low‐cost biomimetic substitute for EHS matrix.^47^ Since collagen is usually derived from animals, its application is limited by similar restrictions as EHS matrix, including variability between batches, insufficient biochemical tunability, and contamination by unknown and xenogeneic components. Likewise, the microstructure of collagen hydrogel (e.g., fibril diameter and alignment) depends on pH and temperature changes during gelation process.^41^ Uncertain collagen fibril size between samples severely limits cell–matrix interaction.

## 3 PROGRESS IN CULTURE STANDARDIZATION OF TUMOR ORGANOIDS

### 3.1 Standardization of organoid source sample collection

Advances in the standardization of organoid culture began with organoid‐based studies aimed at identifying intra‐ and inter‐tumor heterogeneity. Roerink et al. selected three untreated colorectal cancer (CRC) patients and used tumor biopsies from four to six different sites to culture a total of 39 organoid strains, which were subjected to genome‐wide sequencing to generate phylogenetic tree of somatic mutations, revealing the extent of intra‐tumor genetic diversity.^48^ They also revealed that tissues collected from single tumor region failed to illustrate spatial heterogeneity.^48^ Tissue samples from multiple sites or sub‐regions will be able to establish more accurate tumor organoid models. Kopper et al. established a multi‐point organoid model by sampling the primary sites and multiple metastatic sites of an ovarian cancer patient, and analyzed genomics, transcriptomics, morphology, and pharmaceutical response of organoids.^28^ Using a similar approach, Vlachogiannis et al. demonstrated that colorectal and esophageal cancer organoids mimic intra‐ and inter‐tumoral heterogeneity in drug senstivity.^17^

Although tissue collection for organoid generation has not yet been fully normalized, advanced procedures have broken technical bottlenecks. Walsh et al. recovered organoids from cryopreserved breast cancer tissues and highlighted the double‐edged effect of processing time.^49^ Organoids derived from quick‐frozen tissues thawed after 6–12 months of storage had similar drug response profiles compared with organoids constructed from fresh samples of the same tissue origin.

### 3.2 Standardization of organoid construction process and culture environment

**FIGURE 2:**
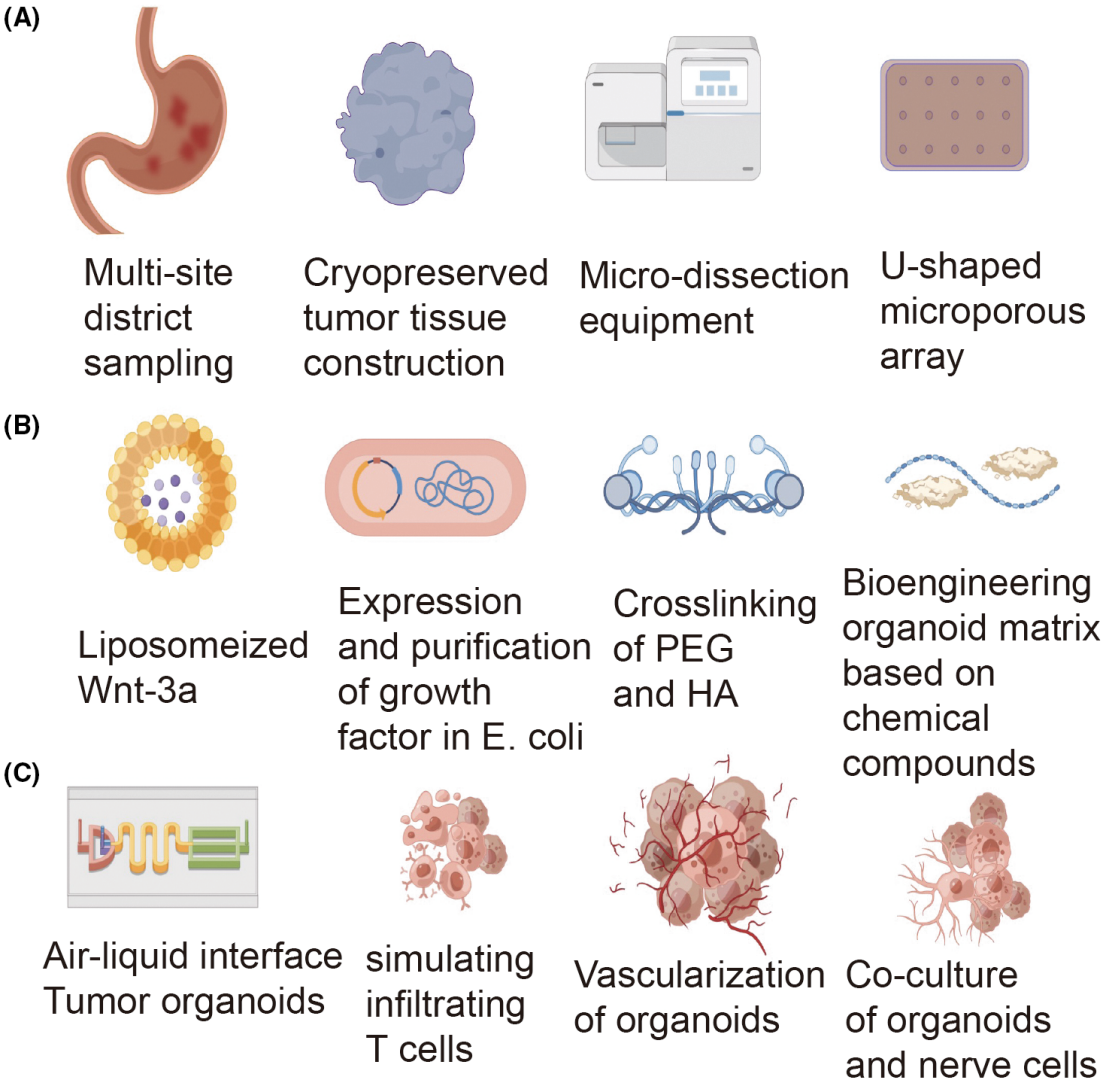
Standardization of tumor organoid construction. (A) Primary and metastatic tumor biopsies along with cryopreserved tumor tissues are all available for organoid construction. Microdissection equipment and U‐shaped microporous array make contributions in processing biopsies and simulating physiological environment in vivo. (B) Progress in organoid medium formulate the application of bioengineering in improving extracellular matrix characteristics. Liposomized Wnt‐3a facilitates its activity, growth factors are expressed and purified in *Escherichia coli*. (C) Progress of organoid culture in TME. Novel techniques (i.e., organoids‐on‐a‐chip) provide a platform for co‐culture of organoids with cells in TME including immune cells. The figure is drawn by Figdraw (www.figdraw.com). TME, tumor microenvironment.

To standardize organoid construction from tumor fragments, Horowitz et al. developed a microdissection procedure to generate millimeter‐scale cuboid tissue sections, improving the uniformity of tissue cutting compared with traditional tissue section techniques.^50^ Using this method, 88% of glioma cuboids were generated in the desired size range (~300–600 μm), and further investigation confirmed that these tissue blocks retained the native TME (Figure 2).

In terms of the number of cells seeded, Brandenberg et al., invented a novel U‐shaped microwell, which customizes the initial cell count to construct healthy gastrointestinal tract and CRC organoids.^51^ In artificial microwells, automated imaging techniques showed a uniform increase in organoid size and morphology.^50^ Li et al. showed 3D air–liquid interface (ALI) cultures could support primary organoid generation, carcinogenic transformation, and long‐term in vitro culture of murine gastrointestinal tissue fragments.^52^ In this culture system, tissues were embedded in a 3D matrix placed on top of the permeable membrane of an inner transwell dish, and the medium was added to the outer Petri dish, allowing nutrients to diffuse through the underlying membrane while exposing the top of the matrix to air. Compared with liquid culture, oxygen transfer efficiency is improved.^53^ Tumor organoids containing both epithelial cells and mesenchymal cells can be fabricated by culturing tissue fragments with this system.

#### 3.2.1 Standardization of organoid culture medium

Tumor organoid should maintain reproducibility for clinical translation. To meet this requirement, production, and purification of growth factors should conform to standardized platform. Tüysüz et al. introduced phospholipid‐ and cholesterol‐based liposomes to enhance the stability and activity of recombinant Wnt‐3a.^54^ Compared with detergent‐based solubilization, liposomal Wnt‐3a enhanced expansion of normal duodenal organoids, supporting serum‐free culture of intestinal and liver organoids.^36^ For medium components with less protein purity, recombinant protein expression methods (e.g., based on bacterial and insects) may be more controllable by performing higher throughput assays. A conspicuous disadvantage of using bacterial platforms is the lack of protein folding and post‐translational modification to maintain protein bioactivity. To address this problem, Urbischek et al. developed a unique method to purify R‐spondin 1 and Gremlin‐1 in *Escherichia coli*.^55^ Both proteins are indispensable for folding and configuring disulfide bonds in specific environments. Disulfide C isomerase and target proteins were co‐expressed in *E. coli* for in vitro disulfide recombination.

In addition to incorporating recombinant proteins into the medium, the genetically engineered agonist of tumor‐related signaling pathway was designed as a cost‐effective substitute with similar biological activity. Janda et al. developed water‐soluble surrogate Wnt agonists by inviting Frizzled‐LRP5/6f heterodimerization and activating downstream beta‐catenin signaling pathways.^56^ In subsequent studies, they designed a “next‐generation surrogate (NGS)” Wnt which could produce the similar level of downstream signaling at a concentration 50‐fold lower than that of the previous generation.^57^ NGS Wnt outperforms Wnt3a conditioned medium in organoid expansion and single‐cell organoid outgrowth.^57^ These surrogates bind to Frizzled and related LRP5/6 receptors and limit their degradation, independent of natural interactions of LGR proteins.^58^

#### 3.2.2 Standardization of organoid ECM

The present biomaterial platform provides favorable conditions for three‐dimensional culture of primary tumor tissue and extends the research of tumor biology and tumor therapy.^59^, ^60^ Xiao et al. encapsulated patient‐derived GBM tissue in a hybrid material consisting of synthetic polyethylene glycol (PEG) modified with an arginine–glycine–aspartate (RGD) integrin protein‐binding peptide and cross‐linked with recombinant hyaluronic acid (HA), an extracellular polysaccharide typically upregulated in GBM.^61^ Compared with conventional glioma spheroid suspensions, GBM organoids grow in high HA content hydrogels and upregulate expression of CD44, which is an HA‐binding cell surface receptor and tumor stem cell marker. Interestingly, GBM organoids cultured in engineered hydrogels with low HA content were approximately three times more sensitive to anti‐tumor drugs than that in stiffness‐matched matrices with high HA content, and knockout of CD44 could counteract this resistant phenotype. These results demonstrate that adjustable matrix platforms facilitate organoid phenotyping and drug sensitivity analysis. However, engineered matrices have not been routinely applied to human organoid culture.^61^

Polyethylene glycol‐based synthetic matrices have been conceived for the culture of pluripotent stem cells and healthy intestinal organoids.^62^ Gjorevski et al. described a PEG‐based synthetic matrix for culturing purified Lgr5+ intestinal stem cells (ISCs), they used a modular synthetic hydrogel network to identify key ECM parameters that control ISC expansion and organoid formation, and showed that different mechanical environments and ECM components are required at different stages of the process. High matrix stiffness (~1300 Pa) greatly enhanced ISC expansion through a yes‐associated protein 1 (YAP)‐dependent mechanism. In contrast, ISC differentiation and organoid formation required a soft matrix (~190 Pa) and laminin‐based adhesion.^47^ Cruz‐Acuña et al. also revealed that synthetic matrix was beneficial to intestinal organoids derived from human pluripotent stem cells, organoids grown in PEG‐based matrix would differentiate into mature intestinal tissues after in vivo injection.^63^ Hernandez Gordillo et al. designed similar synthetic ECM with tunable biomolecular and biophysical properties to identify the gel components that support primary human intestinal and endometrial organoids formation starting from single cells rather than tissue fragments.^64^ They identified the integrin α2β1 ligand GFOGER grafted to an 8‐arm PEG macromer as a critical component governing epithelial cell proliferation and organoid formation in PEG‐based synthetic ECM, while bulk mechanical properties did not appear to be the dominant parameter for human enteroid/organoid growth.^64^

While pure PEG‐based systems offer advantages for adjustable hydrogels, they typically suffer from high matrix swelling and lack of structural features at cellular level. To remedy the deficiencies, researchers are dedicated to biopolymer‐based matrices. Intestinal organoid engineering matrices based on purified silk proteins and alginate polysaccharides are suitable alternatives to animal‐derived matrices.^65^, ^66^ Fibrin gels, purified from human plasma fibrinogen, are animal serum‐free matrices with adjustable stiffness and chemical function. Broguiere et al. pointed out that fibrin gels were suitable for both mouse and human epithelial organoids when supplemented with purified laminin.^67^

### 3.3 Standardization of TME in organoids

The development of a culture platform that mimics in vivo TME heterogeneity and cell–cell interactions has become a research hotspot. Different organoid culture methods can mimic the immune microenvironment of tumors in vitro, and currently include two main models: the reconstituted TME model and the holistic native TME model.

The reconstituted model generally uses Matrigel‐coated culture method, that is, after tumor tissue is separated into single cells or tiny clusters of cells by means of physical splicing or enzymatic hydrolysis, organoids were cultured in ECM, and exogenous immune cells (usually derived from autologous peripheral blood or tumor tissue) were isolated and subsequently co‐cultured with the organoids. In this type of culture, factors such as WNT3A, R‐spondin, epidermal growth factor, and Noggin, a bone morphogenetic (BMP) inhibitor, are commonly used to promote stem cell self‐renewal and differentiation, but different growth factors and/or pathway inhibitors need to be added according to the tissue type subsequently.^10^, ^31^, ^68^ Organoids cultured in this way are highly consistent with the original tumor tissue at both the genetic and pathological levels, so they can be used for in vitro disease modeling and drug screening, and can functionally mimic the response of tumor patients to clinical treatment. However, this culture method of completely immersing organoids does not preserve stromal cells,^9^ so exogenous addition of immune cells is required to build the TME. Currently this reconstruction model has been used in a variety of tumor‐related studies.^10^, ^69^, ^70^, ^71^ After co‐culture of pancreatic ductal adenocarcinoma (PDAC) organoids with tumor‐associated fibroblasts (CAFs) using a Matrigel‐coated culture method, Seino et al. found that Wnt signaling proteins secreted by CAFs can drive organoid growth. In this study, a library of 39 patient‐derived PDAC tumor organoids was established, and three functional subtypes were identified based on their dependence of the stem cell microenvironment on Wnt and R‐spondin.^10^

Öhlund et al. co‐cultured pancreatic stellate cells with PDAC organoids, and identified two distinct CAF subsets (i.e., myofibroblast and inflammatory fibroblast) that exhibited heterogeneity in the organoids, each of which performed a unique function.^70^ Co‐culture of PDAC organoids with CAFs found that tumor‐secreted interleukin‐1 and transforming growth factor‐β promote distinct inflammatory and myofibroblast CAFS subtypes, respectively.^71^ The researchers also used a matrix‐coated culture method to reconstruct organoids with a variety of immune cells. When patient‐matched CAFs and peripheral blood lymphocyte were co‐cultured with PDAC organoids, myofibroblast‐like CAFs are activated, lymphocyte infiltrate into Matrigel and migrate to tumor organoids. This study is the first to report disease‐relevant 3D in vitro models representing pancreatic tumor, stroma, and immune components, co‐cultured using primary tumor samples representing the TME and is expected to facilitate the study of tumor–stroma and tumor–immune interactions and the evaluation of immunotherapy drugs.^69^ Chakrabarti et al. co‐cultured cytotoxic T lymphocytes (CTLs) with bone marrow‐derived dendritic cells activated by tumor antigens released by mouse gastric tumor organoids, and found stimulated CTL had a killing effect on gastric tumor organoids in the presence of PD‐L1 antibodies, demonstrating that the model of co‐culture a variety of immune cells with organoids to mimic the tumor immune microenvironment can effectively study the interaction between tumor cells and immune cells, as well as between immune cells.^72^

Unlike the reconstituted TME model, the holistic native TME model is a unit in which tumor epithelial cells are cultured with stromal endogenous immune cells as a whole, mainly including ALI culture and microfluidic 3D culture. The ALI culture method is to physically cut tumor tissue containing immune cells into tissue fragments and culture them in transwell coated with collagen gel. The top of the gel is exposed to air to allow the cells to receive an adequate supply of oxygen; the medium in the outer dish diffuses into the inner dish to form a ALI. The ALI method not only preserves the basic genetic characteristics of the original tumor but also preserves the complex cellular composition and structure of TME.

Neal et al. cultured patient‐derived tumor organoids by the ALI method to reproduce the patient's tumor immune microenvironment. The researchers cultured tumor tissue from 100 patients with 28 different disease subtypes in vitro with patient‐derived organoids (PDOs) that preserved the fibrous matrix and immune components inherent in the tumor tissue. Single‐cell immunoassays demonstrated that the model preserves the original tumor T‐cell receptor spectrum and successfully mimics immune checkpoint blockade, playing a key role in driving personalized immunotherapy. In ALI organoid cultures, most tumors can grow in their native state, thus preserving a variety of endogenous immune cells, including T cells, B cells, NK cells, and macrophages. ALI PDOs cultures can accurately reflect histologic and other features of the original tumor within a short period (at least 30 days). The immune component of ALI PDOs declines over time compared to tumor epithelial cells that can be passaged and cryopreserved and does not persist for more than 2 months despite IL‐2 supplementation.^22^ ALI culture method provides an integrated strategy for immune TME modeling in vitro, which can explore the interaction between multiple different cell populations.

Vessel system plays an important role in oxygen transport and nutrient exchange. Fluids, gases, and macromolecules can selectively pass through this barrier. The organoid vasculature is a challenge to be solved.^73^ Among in vitro approaches, vascularization of organoids often by means of controlling artificial microenvironment to achieve self‐organizing. There are endogenous vascular endothelial progenitor cells detected at the beginning of organ development. Cytokines were included into culture medium to support differentiation of endogenous endothelial cells (ECs).^74^ However, central nervous system will not produce endogenous ECs. To address this problem, researchers induce vascular ECs from human pluripotent stem cells and co‐culture them with brain organoids.^75^ The researchers induce brain organoids from neuroectoderm and vessels tissues from mesoderm, respectively. They integrated organoids from both germ layers to form vascularized organoid.^76^ Lutolf et al. exploit a 3D scaffold to induce ISCs forming tubular epitheliums. This mini organ maintains key characteristics of intestinal and it can survive couple of weeks by perfusion liquid in lumens to remove dead cells and catch necessary materials.^77^ The arrival of organoids‐on‐a‐chip system provides a more precise and controllable platform, which sort out the essence from organoids‐ and organs‐on‐a‐chip.^78^ Printing 3D bio‐scaffold can support the self‐assemble of ECs to form hollow tubular structure. Incorporating blood vessels and other organoids into a chip device can promote the integrity of circulatory system. In this way, different chips derived from different tissues can be linked by these microfluidic channels.^79^

The vascularization of organoids in vivo mainly induced by transplanting organoids into hosts. Mansour et al. utilized human embryonic stem cells to differentiate into small brain organoids in vitro and transplanted them into leptomeningeal blood vessels in mice.^80^ The liver vessels have been transplanted and connected with host's vessels, forming a complicated vessels network, they are able to be observed from real‐time imaging.^81^

## 4 FUTURE OPPORTUNITIES FOR TUMOR ORGANOID CULTURE STANDARDIZATION

### 4.1 Future opportunities for organoid formation

As patient‐derived tumor organoids are increasingly being applied in preclinic studies, it is important to fabricate organoids in reproducing tumor heterogeneity. Living organoid biobanks have expanded our understanding of tumor phenotypes.^7^, ^82^ Even though these studies have explored spatial heterogeneity of tumors, applications of organoid models for a thorough inquiry of tumor evolution have not been fully put into use. The development of precision medicine will accelerate the process of organoid standardization and clarify how the initial cell population regulates organoid construction through a certain selection of tumor cells. Accumulating techniques including cellular barcoding and machine learning‐based imaging may facilitate quantitative monitoring organoid expansion at cellular level and its clinical application.^83^, ^84^

### 4.2 Future opportunities for organoid culture medium

The continued development of next‐generation organoid medium for organoid culture requires understanding individual tumor niches and modeling them with standard methods. Current criteria for classifying PDOs according to specific media are mainly based on tumor driver gene and mutation status. Studies have implied the diversity of tumor signaling pathways and the factors that drive tumorigenesis even in the absence of genetic alterations or loss of function.^85^ It is critical to identify cytokines involved in multiple signaling pathways and cell types in the TME, and this can be achieved by single‐cell RNA sequencing and proteomic analysis.^86^ Soluble cytokines as potential substitutes for animal‐derived serum are essential for oncology research. Investigation of differences between healthy and tumor stem cell niches may help to explore the pathogenesis of tumors and the underlying mechanisms required for the formulation of culture media.^87^ Decent description of tumor features prior to organoid culture encourages us to develop suitable medium to better mimic tumor signaling and predict drug response in tumor organoids.

The final determination of soluble factors and their working concentration in medium may rely on their physical and chemical properties, which need to be fully explored. Hypoxia is a common condition of tumor TME in vivo, and oxygenation levels in organoid cultures have not been extensively studied. Due to the distribution of various cell types and changes in the vasculature, there is a spatial heterogeneity in the concentration of soluble factors and pH values within the TME.^41^

Traditional tumor organoid culture (i.e., immersion in culture medium) cannot accurately explain this heterogeneity and its impact on tumor phenotype. Thus, innovative technological platforms to control the composition of tumor organoid cultures at the temporal and spatial levels should be important. In addition to microfluidic‐based approach, emerging micromachining technologies (e.g., two‐photon mode) enable us to visualize four‐dimensional models of active growth factors.^88^ It is essential to clarify media components to maintain non‐tumor cell growth and support heterotypic cell interactions.

### 4.3 Future opportunities for organoid ECM

An important goal of biomaterial community is to develop precision‐engineered material platforms to explore minimum requirements for matching the biological output and efficiency of EHS matrices. Although substantial progress has been made, the efficiency of organoid culture in engineered matrices is generally lower than that in EHS matrices.^89^ The low efficiency of engineered matrices may be attributed to their limited biodegradability, reconfigurability, and relatively infrequent incorporation of ECM components and cell‐interacting ligands. The future of polymer and material engineering disciplines should address these limitations while accumulating general ease of use and availability of materials.^53^

Another limitation of engineering matrices comes from the lack of sufficient spatiotemporal control over biochemical and mechanical properties to mimic dynamic TMEs. Several groups have developed platforms that attempt to modify matrix cues in space and time.^88^, ^90^, ^91^ Hushka et al. reported a photodegradable hydrogel platform to study the effects of matrix mechanical properties on intestinal organoid differentiation, and they used controlled photodegradation to promote intestinal organoid differentiation by forming intestinal crypts, the size and number of which depend on the degree of the matrix softening.^92^

The control of spatial organization is achieved by embedding stem cells into injectable matrices by bioprinting.^93^ In parallel with material development, technological advances in detecting the interaction of tumor cells with dynamic ECM are essential to better understand their biological roles. Krajina et al. developed a real‐time, non‐invasive light scattering microfluidic technology to simultaneously detect cell‐mediated matrix flow and solidification processes in an in vitro 3D breast tumor model.^94^ Although several different cell types have been studied, few literatures have so far incorporated this technology into organoid culture.

## 5 CONCLUSIONS

Breakthroughs in tumor biology have been driven by fundamental research and advanced approaches of primary tumor cell culture. Consummate establishment of the first human cancer cell culture, Henrietta Lacks' cervical tumor cells, has expanded our vision to study tumor tissues in vitro. Single‐cell sequencing technology provides unprecedented resolving power, which enables us to deeply explore the cell composition and phenotype of malignant tumors. Tumor organoid culture has further deepened our insight into biological heterogeneity across tumor subtypes. These technological advances allow us to explore more unrevealed issues about tumor heterogeneity, and the successful solutions depends on continued technological improvements and elevated reproducibility.

Each tumor model has inherent limitations in reflecting tumor characteristics and an appropriate tumor model is the basis for successful application on the bench and bedside. With the in‐depth understanding of tumor drivers and treatment approaches, we should pay more attention to the standardization of in vitro tumor models. In this review, we highlight existing limitations of organoid culture, introduce rising approaches for standardization of tumor organoid culture, and present an outlook for applying organoids to recapitulate tumor heterogeneity. The successful construction of organoid model under standardized rules will accelerate its application in translational medicine and precision medicine.

## AUTHOR CONTRIBUTIONS

**Changchun Zhou:** Conceptualization (equal); writing – original draft (equal); writing – review and editing (equal). **Yuanbo Wu:** Writing – original draft (equal); writing – review and editing (equal). **Zeyu Wang:** Writing – original draft (equal); writing – review and editing (equal). **Yanli Liu:** Investigation (equal); writing – review and editing (equal). **Jiaqi Yu:** Investigation (equal); writing – review and editing (equal). **Weiping Wang:** Formal analysis (equal); writing – review and editing (equal). **Sunrui Chen:** Resources (equal); writing – review and editing (equal). **Weihua Wu:** Resources (equal); writing – review and editing (equal). **Jidong Wang:** Visualization (equal); writing – review and editing (equal). **Guowei Qian:** Conceptualization (supporting); writing – review and editing (equal). **Aina He:** Conceptualization (lead); writing – review and editing (equal).

## FUNDING INFORMATION

This work was supported by the National Natural Science Foundation of China (Grant No. 82173358).

## CONFLICT OF INTEREST STATEMENT

The authors declare no conflict of interest.

## Data Availability

The data that support the findings of this study are available from the corresponding author.
